# All About Multiparametric MRI Evaluation in Biliary Tree Complications After Liver Transplant

**DOI:** 10.3390/diagnostics16010093

**Published:** 2025-12-27

**Authors:** Adrian Dumitru Dijmărescu, Cristina Dumitrescu, Cristina Alexandra Nicolae, Robert Mihai Enache, Ioana Gabriela Lupescu

**Affiliations:** 1 Department of Radiology, Medical Imaging and Interventional Radiology I, “Carol Davila” University of Medicine and Pharmacy, 020021 Bucharest, Romania; cristina.dumitrescu@umfcd.ro (C.D.); robert-mihai.enache@drd.umfcd.ro (R.M.E.); 2 Fundeni Clinical Institute, 022328 Bucharest, Romania; cris_nicolae@hotmail.com

**Keywords:** liver transplantation, biliary tree complications, MPMRI, hepatospecific contrast material

## Abstract

**Background/Objectives**: To present, discuss, and illustrate the role of multiparametric magnetic resonance imaging (MPMRI) in the evaluation of biliary tree (BT) complications after liver transplantation (LT) as an integrated part into the multidisciplinary team approach for personalized patients’ treatment. **Methods**: We retrospectively analyzed the MPMRI findings of 317 patients out of 1080 cases with LT, admitted to the Fundeni Clinical Institute from January 2005 to June 2025, who developed biliary complications. **Results**: Biliary complications after LT evaluated by MPMRI included anastomotic strictures in 235 cases (74%), intra- or extrahepatic bile leaks/biloma in 56 patients (18%), secondary cholangitis due to pyogenic cholangitis in 91 cases (29%), liver abscesses in 23 patients (7%), BT lithiasis in 27 patients (8.5%), disease recurrence in 26 cases (8%), and extrinsic BT compression in 1 case (0.3%). **Conclusions**: MPMRI plays a crucial role for the evaluation of BT complications, with the protocol being optimized in correlation with the clinical question or suspicion and with the clinical status of the patient.

## 1. Introduction

**Figure 1:**
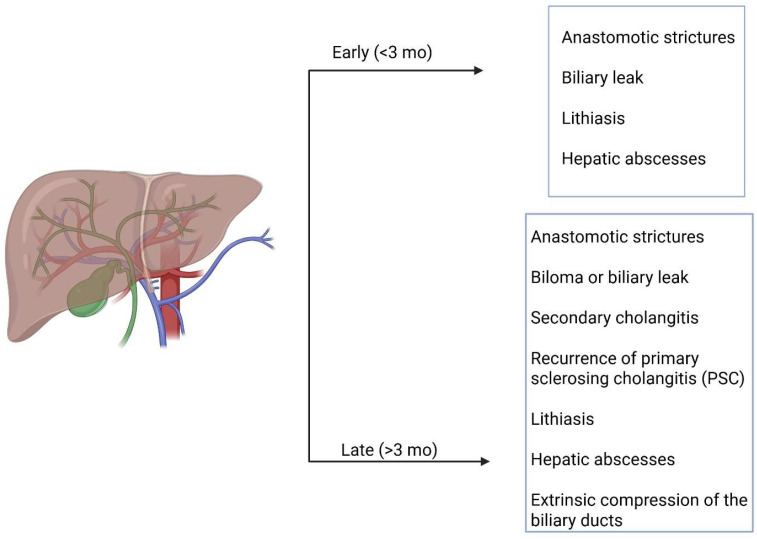
Classification of biliary complications occurring after LT.

Complications occurring after liver transplantation (LT) can be classified according to the time of onset, such as early (<3 months) or late (>3 months), or according to their cause, including surgical (vascular, biliary, or parenchymal), graft-related, immunologic, infectious, and neoplastic, as illustrated in Figure 1 [1,2,3,4,5]. Biliary tree (BT) complications represent the most frequent post-LT complications, with an incidence of 5–25%, particularly in patients undergoing right-lobe living donor transplantation, and they typically occur during the first postoperative month [1,6,7]. The most common causes of BT complications after LT are hepatic artery thrombosis or stenosis, accounting for 90% and 70% of cases, respectively [6,8,9,10]. BT complications represent a major cause of morbidity and mortality among LT patients, with an incidence of 10–32% [1]. These complications primarily include anastomotic strictures, bilomas, secondary cholangitis, hepatic abscesses, lithiasis, recurrence of primary sclerosing cholangitis (PSC), and extrinsic compression of the biliary ducts, all of which significantly impact patient survival and the need for retransplantation [1,3,11]. The most frequent biliary complication is biliary obstruction caused by anastomotic or non-anastomotic strictures (biliary ischemia, pretransplantation biliary disease and infection, papillary dyskinesia due to devascularization/denervation of the ampulla of Vater, or biliary stones) [8,9,10].

The use of ultrasound as an initial investigation may help to identify features suggestive of complications, such as hepatic arterial thrombosis, fluid collections, and biliary tree dilatation. Computed tomography, more widely available than magnetic resonance imaging (MRI), is able to provide detailed cross-sectional anatomy, though it is less comprehensive than MRI, and multiparametric magnetic resonance imaging (MPMRI), especially in current practice—often as complementary methods—can help identify these complications, enabling appropriate patient management [1,5,12]. Endoscopic retrograde cholangiopancreatography (ERCP) has been frequently used to identify biliary leakage but may lead to retrograde cholangitis and pancreatitis. Today, ERCP is the treatment of choice for biliary strictures after their identification by MRI, offering good short- and long-term results [13,14]. Magnetic resonance cholangiopancreatography (MRCP) is currently the gold standard for the non-invasive diagnosis of intra- and extrahepatic biliary complications, while complementary MPMRI sequences—described in detail in the following subsections—support identification of subtle abnormalities and provide a reliable diagnosis (sensitivity and specificity of 98–99% and 94–96%, respectively, as reported by other studies) [1,15,16,17,18,19]. A crucial aspect when assessing BT complications by MRI is understanding the surgical technique (orthotopic implantation of deceased donor grafts, split or reduced deceased donor allografts, living donor segmental LT) and the type of biliary anastomosis—considered the “Achilles tendon” of LT—which is selected based on donor and recipient anatomy and surgeon preference. The most common anastomosis is duct-to-duct (end-to-end or end-to-side), followed by choledochojejunostomy (preferred in patients with pre-existing biliary disease, size mismatch between donor and recipient ducts, retransplantation, or previous biliary surgery) and choledochocholedochostomy (associated with a higher risk of biliary leakage and cholangitis) [1,8,20,21,22,23].

The present study aims to demonstrate and illustrate how a correct and complete MRI protocol in correlation with a systematic analysis of MRI images can help radiologists to recognize subtle imaging features essential for the rapid and accurate diagnosis of BT complications after LT, thereby strengthening their contribution within the multidisciplinary team managing LT patients.

## 2. Materials and Methods

This article presents a retrospective, descriptive, illustrative, and educational study focused on the MRI evaluation of patients who underwent LT and developed biliary complications. We identified 1080 patients with LT between January 2005 and June 2025 admitted to the Fundeni Clinical Institute. The patients were aged between 1 and 70 years with a median age of 52 years, with a male/female ratio of 1.4. Out of all LT patients, we included in the study group 317 patients (29.35%) who developed biliary complications. Routine MRI surveillance was not performed in all transplanted patients. Multiparametric MRI examinations were performed in patients with either suggestive clinical symptoms or cholestasis on laboratory tests, or inconclusive ultrasound and/or computed tomography findings raising suspicion of biliary complications. The main clinical signs of biliary complications were right upper quadrant pain, fever, and nausea or vomiting. Biliary complications occurred in the early phase after LT, in the first three months, or in a late phase for the majority during the first year. All the information was collected from the Department of Radiology, Imaging, and Interventional Radiology in accordance with patients’ written consent recorded in the hospital admission sheet.

Inclusion criteria were availability of complete multiparametric MRI examinations and confirmed imaging diagnosis of BT complications. Patients with incomplete imaging studies or insufficient clinical data were excluded from the analysis. The final diagnosis of biliary complications was based on MRI findings in combination with clinical and laboratory data.

MRI is the preferred imaging technique for BT evaluation when ultrasound and/or computed tomography are inconclusive or as a follow-up method when biliary complications are known and therapeutic management is conservative [1,24,25,26,27]. In our practice, MPMRI plays a central role for the non-invasive evaluation of BT complications, with ERCP being primarily used for therapeutic interventions. Therefore, statements regarding MRI are reflective of its clinical importance in our center, rather than a formal comparison with other diagnostic methods. In all cases with clinical, laboratory data, and ultrasound evaluation in favor of anastomotic stenosis without or in association with biliary lithiasis, the MRI protocol using 1.5 and 3 T equipment, as shown in Table 1, and included coronal 3D MRCP, coronal, oblique, and axial SSFSE short TE T2wi acquisitions with thin slices, axial T2 Fat Sat wi, DWI with ADC map [25,26,27], and unenhanced 3D T1FatSat. In all patients with clinical suspicion of anastomotic stenosis associated with signs of inflammation, biloma, and/or liver disfunction, the MPMRI protocol included, in addition to the sequences mentioned above, 3DT1FatSat before and after hepatospecific gadolinium-based contrast material (HSCM) i.v. injection, using a multiphase acquisition (in late arterial, portal, and transitional phase and hepatobiliary phase). We used between 2005 and 2010 gadobenate dimeglumine (Gd-BOPTA ((Multihance) Bracco, Mailand, Italy)) 0.1 mL/kg with the achievement of the hepatobiliary phase two hours or even more post-i.v. injection due to reduced biliary excretion of this HSCM (only 2–4% of the administered dose is taken by the liver cells). After 2010, we used gadoxetic acid (GD-EOB-DTPA ((Primovist) Bayer Healthcare, Berlin, Germany)) 0.1 mL/kg, with the hepatobiliary phase being accomplished depending on the specific laboratory data (total bilirubin, direct bilirubin, indirect bilirubin), and MRI findings between 20 and 60 min or even later in severe obstructive jaundice (50% of GD-EOB-DTPA injected dose is biliary excreted).

In few cases, for which the clinical suspicion was either severe obstructive biliary stricture or biloma, we performed a short MPMRI protocol, as shown in Table 2, that consists of injecting 10 mL of contrast agent before entering the MRI room, followed by a waiting period of 7–10 min. The patient is then positioned in the scanner, localization images are acquired, and the following sequences are performed: coronal and oblique SSFSE short TE T2-weighted imaging, 3D MRCP, axial T2 Fat Sat imaging, and DWI with ADC map and axial and coronal 3D T1 Fat Sat, representing the hepatobiliary phase [28,29,30].

For all MRI evaluations, we analyzed the aspect of the bilio-biliary or bilio-digestive anastomoses and the degree of stenosis, the aspect of the intrahepatic BT (signs in favor of acute or chronic inflammation, degree of dilatation, presence of biliary lithiasis or pus, degree of BT walls enhancement, presence of satellite BT abscesses), presence of biliary leak, or of other focal liver lesions, the aspect of the liver parenchyma pre- and post-HSCM, in correlation with diffusion, T2 FS, T2 GRE, T1 with TE in -/out of phase wi, taking into account that gadoxetic acid is considered a biomarker for the liver and biliary tree, allowing the extraction of the morphological changes and also functional alteration such as liver disfunction.

Descriptive statistical analysis was performed to summarize the frequency and distribution of biliary complications. Results are expressed as absolute numbers and percentages. Comparative analysis between OLT and LDLT is descriptive only, given the retrospective design of the study; no inferential statistical tests or confidence intervals were calculated. No control group or formal analytical comparison was performed.

**Figure 2:**
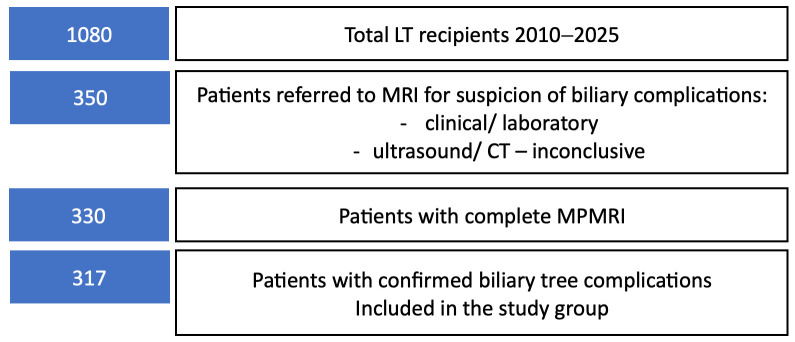
Flow diagram illustrating the selection of liver transplant recipients included in the study.

Patient selection for multiparametric MRI evaluation is summarized in Figure 2.

## 3. Results

### 3.1. Pre-Existing Liver Disease

Knowledge of the pre-existing condition of the patient before LT and the pathologic findings in the explanted liver will guide the postoperative surveillance, including imaging. Dominant diseases as indication for LT were hepatocarcinoma developed on viral or alcohol-induced cirrhosis (249 cases), B virus with or without delta virus cirrhosis (449 cases), alcohol-induced cirrhosis (170 cases), C virus cirrhosis (208 cases) followed by PSC (26 cases), autoimmune hepatitis (21 cases), Budd Chiari disease (17 cases), Wilson disease (36 cases), acute fulminant hepatitis (4 cases), and Caroli disease (6 cases).

### 3.2. Liver Transplant Surgical Technique

Knowing the different types of reconstruction that occur during LT is mandatory when we perform an MPMRI evaluation in a patient with a potential biliary complication. In orthotopic LT (OLT), the biliary anastomoses were duct-to-duct end-to-end in 940 patients and hepatico-jejunal end-to-side in 140 cases.

### 3.3. Biliary Complications After LT

Biliary complications after LT were identified by imaging in 317 patients (Table 3).

Some patients experienced more than one biliary complication; therefore, reported percentages may sum to more than 100%.

Both OLT and living donor LT (LDLT) grafts were affected in different percentages (Table 4).

Abscess formation was identified as a complication of cholangitis secondary to anastomotic stricture.

In 24 cases followed up for anastomotic stricture, we discovered tumoral recurrence of hepatocarcinoma.

In the studied group, 21 patients needed retransplantation because of graft failure biliary complications being the cause in 5 (24%) of the explanted liver, 3 patients developed secondary cholangitic cirrhosis, and 2 had PSC with progression to cirrhosis.

Bile leaks and biloma occurred predominantly in the early post-transplant period, except for cases in which biloma developed secondary to late hepatic artery thrombosis, anastomotic strictures occurred quite equally in the early and late phase, stone formation, non-anastomotic strictures due to secondary cholangitis, liver abscesses, and PSC occurred in the late phase.

### 3.4. Biliary Anastomotic Stricture

The incidence of biliary anastomotic strictures was 74% in all LT affecting more the OLT (74% vs. 66% in LDLT).

**Figure 3:**
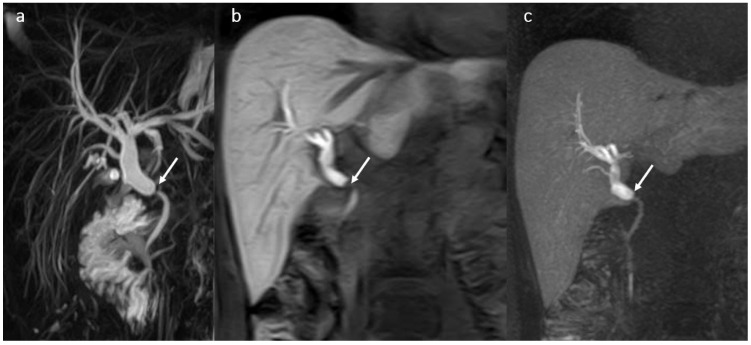
Biliary anastomotic stricture (arrow) in a male patient 37 years old, 1 year after OLT: anastomotic stricture with proximal dilatation of the BT-MRCP (**a**), 3DT1 FS with gadoxetic acid in hepatobiliary phase in coronal plane (**b**) and MIP (**c**).

Imaging was performed in most cases due to signs of cholangitis such as fever, right upper quadrant pain, nausea with or without vomiting, and only rarely in patients who were asymptomatic but had a cholestatic pattern on laboratory tests. Multiparametric MRI with MRCP and HBP after HSCM i.v. injection was the method of choice for assessing the BT abnormalities (Figure 3).

Stricture recurrence and continued stricture formation are possible even after successful therapy. Long-term observation with MRCP and laboratory tests of these patients are required to evaluate the disease course and response to treatment.

### 3.5. Biloma and Bile Leaks

Bile leaks/biloma occurred mainly in the early phase, more frequent in the first 30 days after LT, and were located outside the liver parenchyma in the vicinity of the bile leak (50 cases), or rarely (6 cases) in the liver parenchyma due to late hepatic artery thrombosis.

In our study, the incidence of bile leaks with or without biloma was 15% for OLT and 19.5% for LDLT.

**Figure 4:**
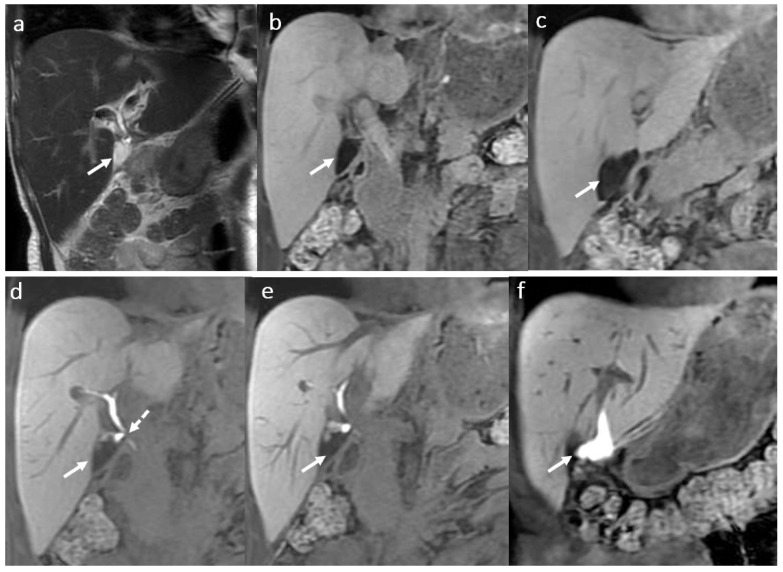
Biloma in a male patient 35 years old, 2 weeks after OLT: T2-hyperintense (**a**) and T1 FS hypointense (**b**,**c**) fluid collection (arrow) adjacent to the biliary anastomosis, with late hepatospecific contrast opacification in hepatobiliary T1FS phase (**e**,**f**) secondary to a biliary fistula ((**d**) dotted arrow).

MRI with HSCM is the best imaging modality using the HBP acquisitions to visualize the bile leak, allowing the optimal therapeutic approach (Figure 4).

**Figure 5:**
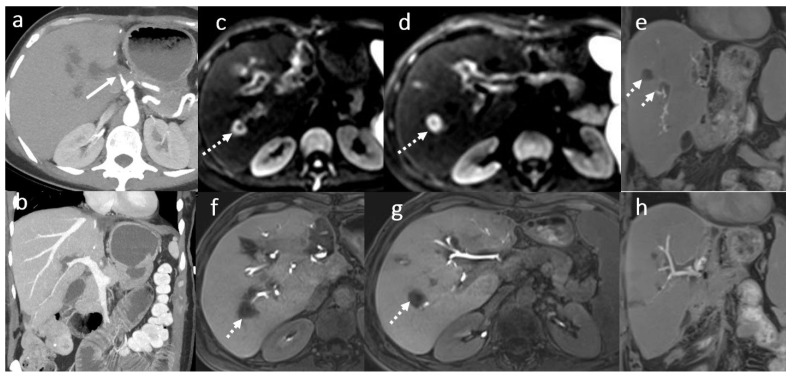
Circumscribed areas of peribiliary liver necrosis due to hepatic arterial thrombosis in a female patient, 49 years old, 3 weeks after OLT: CT angiography (**a**,**b**) hepatic arterial thrombosis (arrow). Fluid collection around some of the subsegmental intrahepatic biliary ducts (dotted arrows) in MRI evaluation using diffusion wi (**c**,**d**) and 3D T1FS with gadoxetic acid in HBP (**e**–**h**).

When imaging fails to depict the leak due to different causes that impair contrast material to be excreted in the right amount through the BT, ERCP and/or percutaneous procedures are used. Bile leaks occur more frequently at the anastomotic biliary site. The use of gadoxetic acid and of the hepatobiliary T1 FS phase allow the possibility to differentiate between intrahepatic biloma and circumscribed areas of peribiliary liver necrosis due to arterial thrombosis (Figure 5).

### 3.6. Secondary Cholangitis

Secondary cholangitis was identified in 25.5% of patients after OLT and in 17% after LDLT. In most cases, the patients had multiple episodes of pyogenic cholangitis (based on suggestive symptoms and laboratory tests) secondary to anastomotic strictures. Acute bacterial cholangitis in chronic cholestasis is challenging as symptoms are atypical.

**Figure 6:**
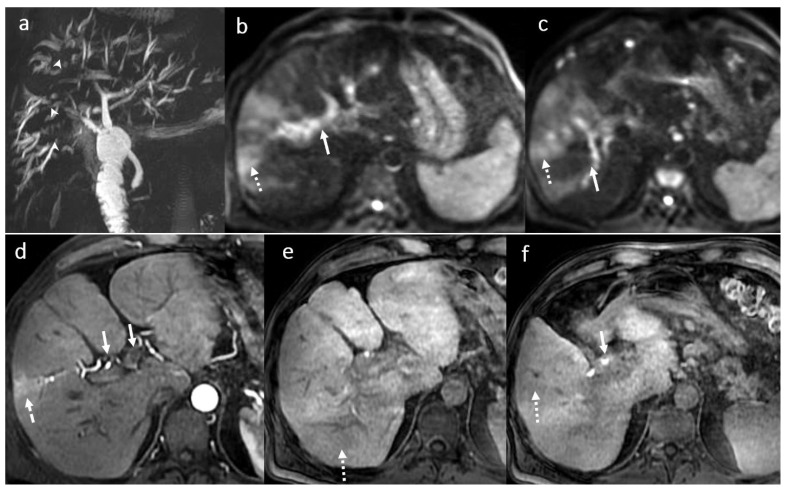
Acute cholangitis in a male patient, 70 years old, 3 months after OLT: multiple intrahepatic focal BT strictures (arrowhead) with mild intrahepatic BT dilatation until the periphery (MRCP) (**a**) in association with inflammatory changes involving the central parts of the intrahepatic BT, the right and the left bile ducts, the main common duct (symmetrical circumferential thickening of the walls (white arrows)) and focal areas of the liver parenchyma (dotted arrow) seen in diffusion wi (**b**,**c**), in arterial (**d**) and hepatobiliary phase (**c**,**d**) after gadoxetic acid i.v. injection (**e**,**f**).

MPMRI examination was the main method of diagnosis for cholangitis, being sensitive to changes of the intrahepatic BT, of the periductal space, and of the liver parenchyma. MRI can depict acute changes of cholangitis by identifying focal bile duct wall thickening with hyperenhancement, purulent bile visible as hyperintense content on DWI and hypointense on the ADC map, periductal and T2 wedge-shaped parenchymal high signal intensity that are correlated with DWI (Figure 6).

In chronic cholangitis, dilatation of the first- and second-order ducts, with non-dilated or non-visualized peripheral ducts (pruning) secondary to stricture/s in the central part of the BT may be present.

**Figure 7:**
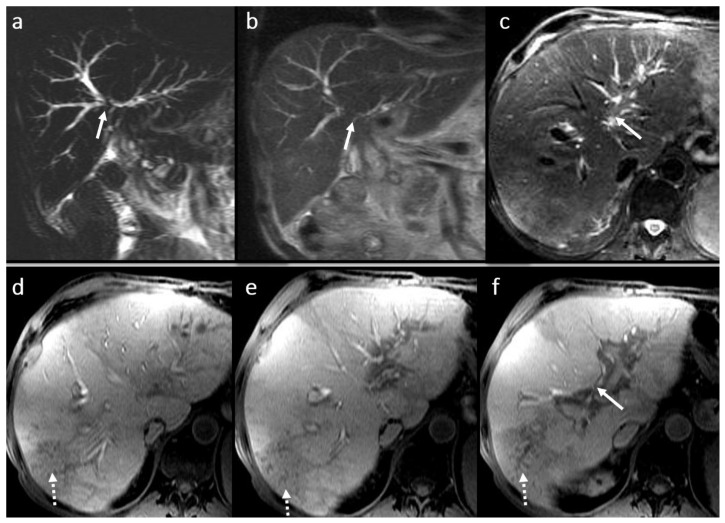
Chronic cholangitis with perianastomotic fibrosis (arrow) in a male patient, 53 years old, 4 year after LT: MRI evaluation in SSFSE long TE (**a**), SSFSE short TE (**b**), T2FS wi (**c**), and 3DT1FS in hepatobiliary phase (**d**,**e**): mild dilatation with discrete irregularities and short multiple focal stenosis of the intrahepatic BT secondary to perianastomotic fibrosis; note also small filling defects in the left intrahepatic bile duct (microlithiasis) in association with peripherical pseudo-triangular hypointense T1 liver areas in HBP corresponding to focal liver disfunction due to biliary obstruction (dotted arrow) (**f**).

Chronic cholangitis was considered if persistence of BT inflammation or multiple recurrent acute episodes of cholangitis exceeded a period of 6 months (Figure 7).

Laboratory tests and symptoms may lead toward a diagnosis, but there are overlaps that impose a differential diagnosis. The presence of a newly identified hepatocellular pattern abnormality associated with cholestasis and fever may be indicative of abscess formation. In our study, 23 patients developed hepatic abscess formation as a complication of acute cholangitis. The main cause of abscess formation after LT is considered to be biliary anastomotic strictures in up to 78.6% of patients. Liver abscess formation occurs mostly in the first year after LT.

### 3.7. BT Lithiasis

**Figure 8:**
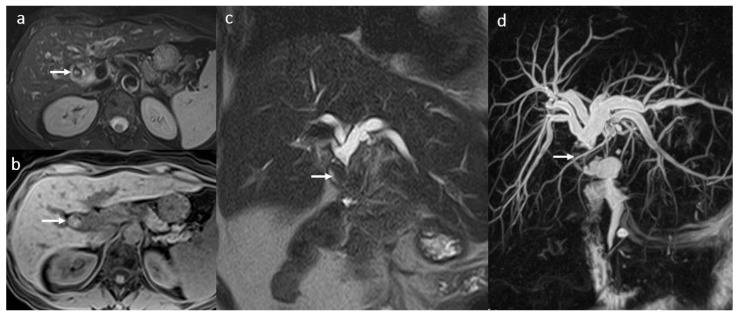
Obstructive biliary lithiasis in a female patient, 58 years old, 2 years after-LT: large round filling defect (arrow) predominantly T2 hypointense (**a**,**c**,**d**), T1 FS hyperintense (**b**), located in the common hepatic duct (arrow) with important BT dilatation clearly visible in MRCP (**d**).

Twenty-seven cases developed BT lithiasis with late onset. In 20 patients, the main cause was obstruction and stasis due to anastomotic biliary stricture. MRCP with short and long TE was the diagnostic tool used in all cases (Figure 8).

### 3.8. Primary Sclerosing Cholangitis

**Figure 9:**
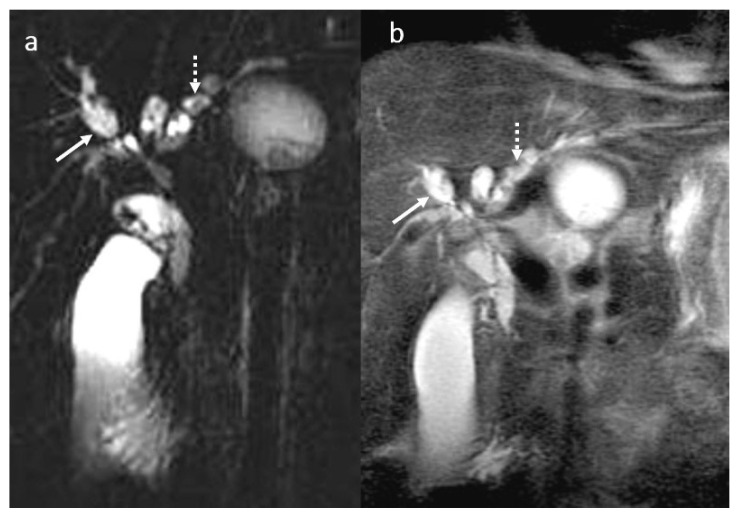
PSC in a male patient, 30 years old. MRI aspect before liver transplant: MRCP (**a**), T2 wi SSFSE short TE (**b**): multiple saccular and fusiform dilatation (arrow) in association with multiple short stenosis involving the intrahepatic and extrahepatic bile ducts with a moniliform appearance. Also note the presence of intrahepatic biliary lithiasis (dotted arrow).

PSC is an immune-mediated disorder characterized by multifocal bile duct strictures, progressive cholestatic disease, and heightened lifetime risks of cancer (Figure 9). In patients with advanced disease, LT is the only life-extending intervention. PSC recurrence is observed in approximately 30% of recipients, leading to graft loss and need for retransplantation. In our study, recurrence of PSC was discovered in 2 patients with OLT with rebound of the disease in the graft after 5 years with evolution to cirrhosis in 10 years with explant and retransplantation of a new liver graft from a deceased donor. MPMRI findings were periportal inflammation with hyperenhancement of the intrahepatic BT walls and peripheral wedge-shaped parenchymal areas with subsequent multiple stricture formations with mild dilatations, periportal fibrosis, and cirrhotic configuration of the liver. The final diagnosis was established by liver biopsy and by histopathological examination of the explanted liver.

**Figure 10:**
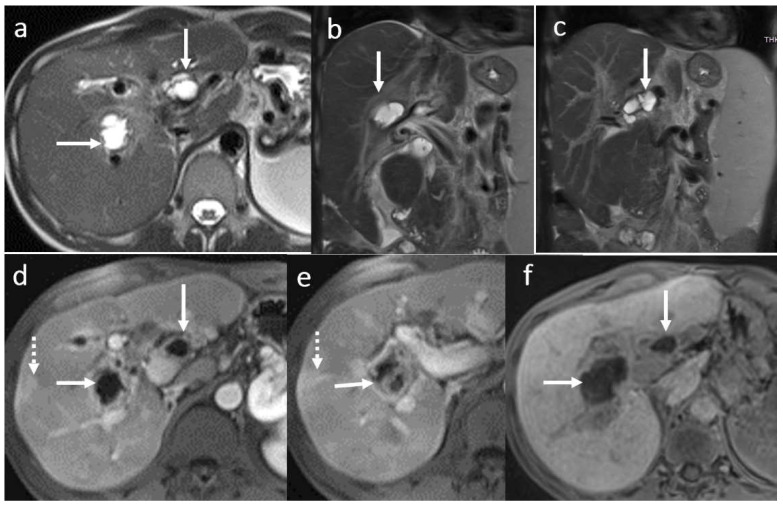
PSC recurrence 8 years after OLT: large saccular centro-hilar BT dilatation (arrows) due to strictures of the right and left bile ducts secondary to thickening, fibrosis, and inflammation of the bile duct walls (**a**–**f**). Liver perfusion abnormality with a triangular shape (dotted arrow (**d**,**e**)). Absence of the HSCM excretion into the centro-hilar part of the BT and extrahepatic bile duct (**f**). Portal hypertension.

In these cases, the role of MPMRI is to detect and assess progression of PSC and complications like calculi, acute bacterial cholangitis, abscess, or cholangiocarcinoma (Figure 10).

### 3.9. Other Changes

**Figure 11:**
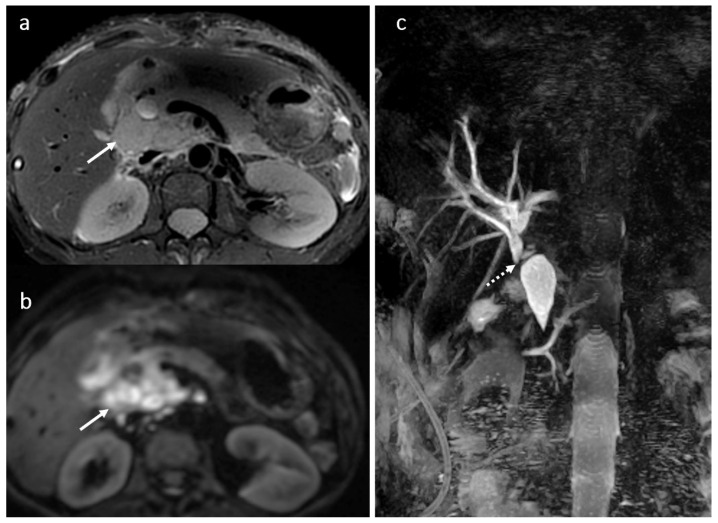
PTLD in a female patient, 49 years old, 2 years after OLT: restrictive tumoral hilar adenopathies with extension between the portal vein and the inferior vena cava (arrow) (**a**,**b**); compression and secondary dilatation of the common biliary duct (dotted arrow) (**c**).

**Figure 12:**
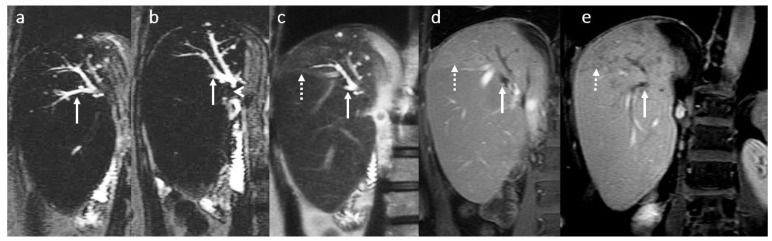
Focal intrahepatic acute cholangitis in a female patient, 70 years old, 2 years after LDLT (right liver lobe from her daughter) with jaundice and fever. MRI evaluation in coronal 2D T2 SSFSE long TE (**a**,**b**) and short TE acquisition (**c**) in association with coronal 3D T1 FS after gadoxetic acid i.v. injection in arterio-portal phase (**d**) and transitional phase (**e**): moderate dilatation of posterior right intrahepatic bile ducts (white arrow) secondary to a tight fibrotic stenosis of the bilio-digestive anastomosis (head arrow) involving the posterior right biliary duct ramifications; mild T2 hyperintensity and enhancement of the adjacent liver parenchyma visible in late arterial phase in association with hypointensity in the transitional phase are suggestive for inflammatory changes (dotted arrow).

During imaging follow-ups for anastomotic biliary stricture, tumoral recurrence of hepatocarcinoma was diagnosed in 24 cases, carrier of the B hepatitis virus after LT, posttransplant lymphoproliferative disorders (PTLDs) in 1 case (Figure 11), and focal intrahepatic acute cholangitis in 1 case (Figure 12).

A comparative analysis showed differences regarding the incidence of biliary complications according to graft type. Anastomotic strictures were more frequently observed in OLT than in LDLT (74% vs. 66%), as shown descriptively. In contrast, bile leaks were slightly more common in LDLT (19.5% vs. 15%). Secondary cholangitis occurred more frequently in OLT (25.5%) than in LDLT (17%). Bile leaks and bilomas predominantly occurred in the early post-transplant period, whereas stone formation, non-anastomotic strictures, secondary cholangitis, liver abscesses, and recurrence of primary sclerosing cholangitis were mainly observed during the late phase after transplantation. These comparisons are descriptive and intended to illustrate trends rather than to infer statistical significance.

## 4. Discussion

The patients with liver graft were transplanted according to the EASLD and Milan and extraMilan criteria [31,32].

Biliary complications were the second cause of retransplantation in our cohort (20%), similar to the incidence reported in the literature [33,34,35].

Liver grafts were obtained from deceased donors or living donors—mostly right lobe and rarely left lobe or dual grafts.

In order to analyze MPMRI findings in transplanted patients it is important to understand the new normal anatomy, namely, the surgical techniques used as anastomoses are the most frequent location of complications [36,37,38].

In this retrospective descriptive study, we did not include inferential statistical analysis; also, we did not aim to compare different types of BT complications after LT or surgical protocols in detail.

The preferred biliary anastomosis is duct-to-duct end-to-end type, and if this is not possible the anastomosis is an end-to-side choledochojejunostomy. In LDLT, the biliary reconstruction is more complex, and adapted to each patient, with end-to-side choledochojejunostomy being the first type used, followed by duct-to-duct end-to-end or combined [36,37,38].

As reported in previous studies, biliary complications typically present with non-specific clinical symptoms (fever, jaundice, nausea, and abdominal pain), which emphasizes the importance of imaging for early and accurate diagnosis. Biliary complications after LT have a high occurrence rate, estimated at 30%. In our center, 29.35% of the patients with LT developed biliary complications. Early diagnosis and prompt management of biliary complications following LT have been shown to reduce morbidity and mortality with improvement of graft survival [34,36,37].

Within current diagnostic algorithms, ultrasound remains the first-line imaging method after liver transplantation; however, its limited sensitivity for defining the cause of biliary abnormalities (estimated at 61%) often requires further evaluation. In this context, MPMRI, combining MRCP, diffusion-weighted imaging, and hepatobiliary phase imaging after gadoxetic acid administration, plays a central role as a comprehensive, non-invasive second-line modality [33,36].

MPMRI is currently considered the imaging technique of choice for the diagnosis of BT complications and its causes, and also for the evaluation of the liver parenchyma. Patency of biliary stent was also evaluated in the presence of cholestasis [33,36].

Compared to Gd-BOPTA, GD-EOB-DTPA is the preferred HSCM used in MRI to evaluate BT complications and liver parenchyma—tight strictures with liver graft disfunction, biloma, acute cholangitis, and suspicion of tumoral recurrence, because of its higher percentage of biliary excretion (50%) and its shorter delay for the hepatobiliary phase (20 min). In our study, in patients with severe cholestatic pattern, we performed the hepatobiliary phase after more than 60 min in order to obtain a good opacification of the BT to help assess changes in caliber and contour of biliary ducts and to evaluate bile leaks, especially at the anastomotic site. Combining 2D and 3D MIP T2W MRCP sequences with 3DT1 HBP increased reader confidence in depicting BT complications. Improvement of accuracy for the assessment of the biliary anatomy by combining 3DT1 HBP with MRCP compared with the use of MRCP alone is considered to reach 88% vs. 69% [39,40].

Furthermore, periportal inflammation and fibrosis associated with secondary recurrent cholangitis or PSC can be accurately depicted using a combination of T2-weighted, diffusion-weighted, dynamic 3D T1 FS contrast-enhanced, and hepatobiliary phase imaging [39,40].

In our study, 91 patients with secondary recurrent cholangitis and 2 with PSC progressed to fibrosis in time and had an indication for a new liver transplant. Patients had multiple MRI examinations during the acute episodes of cholangitis. The one patient who was retransplanted for PSC was further evaluated by MRI according to the guidelines, because the changes in the BT, periportal space, and in the parenchyma were highly suggestive for this diagnosis, considering also that the first liver transplant was for PSC [35].

Bile leaks at the biliary anastomotic site were diagnosed using 3D HBP sequence; in some cases, scanning was after more than 60 min when the precise site of the intrahepatic leak was more difficult to detect. A 90 min 3DT1 HPB acquisition for detection of bile leaks is also proposed by other authors in correlation with the bilirubin level [41,42,43,44].

In all cases, stone formation was diagnosed using MRCP and SSFSE short TE sequences. The sensitivity of the combined interpretation of the MRCP set and the 3D T1 Fat Sat-weighted image is considered to reach 96–97% but may lower in cases with slightly dilated BT and recurrent cholangitis. In case of CBD stones, the sensitivity of MRCP for the diagnosis of common bile duct stones is considered to reach 99%, with the sensitivity for ERCP being reported at 88.5% [45,46,47,48].

In this study, MRI provided detailed non-invasive imaging of biliary complications, complementing clinical assessment. While MRI is valuable for diagnosis and monitoring, ERCP remains the primary therapeutic modality, consistent with current clinical practice.

**Structured MRI report for biliary complications after liver transplantation**. The MRI report must contain the following:

- Indication: the clinical suspicion of the BT complication.
- MRI technique and, if necessary, the type and dose of the contrast material used.
- MRI findings:
  ◦ Bile ducts.
    ▪ Intrahepatic bile ducts dilatation: yes/no and degree of dilatation: mild/moderate/severe.
      • Diffuse.
      • Focal.
    ▪ Aspect and number of strictures: short/ long, unique/multiple.
    ▪ Aspects of the bd content (homogenous/ heterogenous, diffuse/focal) presence of lacunar images suggestive for lithiasis.
    ▪ Aspects of the walls (thickening: yes/no, uniform/asymmetric, regular, irregular), presence of enhancement (acute inflammation).
  ◦ Biliary anastomotic stricture: yes/no and aspect: symmetrical /asymmetrical thickening.
  ◦ Biliary fistula: yes/no.
    ▪ Location of the fistula.
  ◦ Liver parenchymal aspects.
    ▪ Steatosis/ hemochromatosis/ fibrosis.
    ▪ Focal liver lesion(s).
      • Tumoral.
      • Inflammatory.
      • Vascular.
  ◦ Functional liver data in HBP.
  ◦ Other findings:
    ▪ Free fluid in the abdominal cavity.
    ▪ Adenopathies.
    ▪ Other intraabdominal lesions.
- Conclusion: Type of biliary complication and recommendation for future management.

The added value of our study lies in the large patient cohort and the long follow-up period, which allowed a comprehensive assessment of both early and late biliary complications. Moreover, the systematic use of hepatobiliary phase imaging proved particularly useful for differentiating bile leaks, bilomas, and ischemic biliary injury, supporting previous reports while reinforcing its practical relevance in daily clinical practice. The implementation of structured reporting further enhances the clinical utility and educational impact, distinguishing this work from previous pictorial reviews.

This study has several limitations that should be acknowledged. Its retrospective design may have introduced selection bias, and the analysis relied primarily on descriptive and comparative data rather than advanced statistical modeling. In addition, as a single-center experience, the results may reflect local surgical techniques, imaging protocols, and patient management strategies, potentially limiting generalizability to other transplant centers. Changes in surgical techniques, biliary stent use, and MRI technology over the study period may act as confounding factors in frequency analyses. However, as this study is focused on the MRI characteristics of biliary complications, these factors do not affect the main conclusions, which remain descriptive and educational in nature. Nevertheless, the large patient cohort, long study period, and standardized MPMRI protocol provide robust and clinically relevant data that reflect real-world practice in a high-volume liver transplant center.

## 5. Conclusions

MRI plays a crucial role in the evaluation and diagnostic of BT complications, with the protocol being optimized in accordance with the clinical question and the status of the patient.

MPMRI using MRCP with DWI and 3D T1w multiphase acquisition with i.v. injection of gadoxetic acid represents the method of choice for assessing the cause and changes of BT complications such as anastomotic strictures with acute cholangitis, biloma, tumoral recurrence, and liver disfunction.

The MRI structured report contributes to establish a consensus for the multidisciplinary team, for the correct management of each complicated case.

In this descriptive series, MRI allowed detailed visualization of various complications and contributed to clinical decision making without implying formal statistical comparisons with other diagnostic methods.

## Figures and Tables

**Table 1:** MRI protocol for biliary complications after LT.

MRI Sequences	Plane	Notes
Localizer (fast T2)	Axial, coronal and sagittal	For proper positioning.
T2-weighted (SSFSE short TE)	Coronal, oblique (thin slices)	General morphology, edema, fluid, ductal dilatation.
T2-weighted (SSFSE long TE)	Coronal	High sensitivity for bile ducts, bilomas.
3D respiratory-triggered MRCP	Coronal	Projectional images of biliary anatomy, strictures, or leaks.
2D GRE T1 TE in-phase/out-of-phase	Axial	Fat, graft steatosis iron, hemorrhage.
3D T1 FS dynamic contrast enhanced (native, late arterial, portal, and transitional phase)	Axial	Precontrast baseline. Vascular status, ischemia, focal liver lesions characterization.
T2-weighted FS	Axial	Fluid, collections, bilomas, abscesses.
DWI/ADC	Axial	Infection, abscess, rejection, ischemia, detection, and characterization of focal liver lesion.
T2 GRE	Axial	Hemochromatosis, siderotic nodules, calcifications.
3D T1 FS in hepatobiliary phase—20 min post GD-EOB-DTPA (gadoxetic acid) i.v. injection	Axial, coronal	Biliary excretion, anastomotic patency, detection, and characterization of focal liver lesions.
3D T1 FS in late hepatobiliary phase—60+ min post GD-EOB-DTPA (gadoxetic acid) i.v. injection	Axial, coronal	Biliary obstruction with acute cholangitis, liver disfunction, biliary leaks.
3D T1 FS in late hepatobiliary phase—2 h or more post Gd-BOPTA (gadobenate dimeglumine) i.v. injection	Axial coronal	Biliary obstruction with acute cholangitis, liver disfunction, biliary leaks, characterization of focal liver lesions.

**Table 2:** Short MPMRI protocol for biliary complications after LT.

MRI Sequences	Plane	Notes
Localizer (fast T2)	Axial, coronal and sagittal	For proper positioning.
T2-weighted (short TE)	Coronal ± oblique	General morphology, edema, fluid, ductal dilatation.
3D respiratory-triggered MRCP	Coronal	Projectional images of biliary anatomy, strictures.
T2-weighted FS	Axial	Fluid, collections, bilomas, abscesses.
DWI/ADC	Axial	Infection, abscess, rejection, ischemia, detection, and characterization of focal liver lesions.
3D T1-weighted GRE FS in hepatobiliary phase	Axial and coronal	Biliary excretion, anastomotic patency, biliary leaks.

**Table 3:** BT complication after LT.

BT Complications	Percentage of Patients (%)
Anastomotic biliary stenosis	74%
Biloma/bile leaks	18%
Secondary cholangitis	29%
Hepatic abscess	7%
Lithiasis	8.5%
PSC recurrence	0.5%
Neoplasms (recurrence)	7.5%
Extrinsic compression	0.3%

**Table 4:** Incidence of biliary complications depending on graft type.

Biliary Complications	Number of Patients (%) with OLT	Number of Patients (%) with LDLT
Anastomotic biliary stenosis	74%	66%
Biloma/bile leaks	15%	19.5%
Secondary cholangitis	25.5%	17%
Hepatic abscess	6%	7%
Stones	9%	5%
PSC recurrence	0.4%	2.5%

## Data Availability

The original contributions presented in this study are included in the article. Further inquiries can be directed to the corresponding authors.

## Abbreviations

The following abbreviations are used in this manuscript:

LT	Liver transplant
BT	Biliary tree
PSC	Primary sclerosing cholangitis
MRI	Magnetic resonance imaging
MPMRI	Multiparametric magnetic resonance imaging
ERCP	Endoscopic retrograde cholangiopancreatography
MRCP	Magnetic resonance cholangiopancreatography
DWI	Diffusion weighted imaging
HPB	Hepatobiliary phase
HSCM	Hepatospecific contrast material
PTLD	Posttransplant lymphoproliferative disorders
OLT	orthotopic liver transplant
LDLT	living donor liver transplant
GD-EOB-DTPA	Gadoxetic acid
Gd-BOPTA	Gadobenate dimeglumine

## References

[1] Vernuccio F., Mercante I., Tong X.X., Crimì F., Cillo U., Quaia E. (2023). Biliary complications after liver transplantation: A computed tomography and magnetic resonance imaging pictorial review. World J. Gastroenterol..

[2] Daniel K., Said A. (2017). Early Biliary complications after liver transplantation. Clin. Liver Dis..

[3] Rönning J., Berglund E., Arnelo U., Ericzon B.G., Nowak G. (2019). Long-term Outcome of Endoscopic and Percutaneous Transhepatic Approaches for Biliary Complications in Liver Transplant Recipients. Transplant. Direct.

[4] Lemmers A., Pezzullo M., Hadefi A., Dept S., Germanova D., Gustot T., Degré D., Boon N., Moreno C., Blero D. (2021). Biliary cast syndrome after liver transplantation: A cholangiographic evolution study. J. Gastroenterol. Hepatol..

[5] Di Martino M., Rossi M., Mennini G., Melandro F., Anzidei M., De Vizio S., Koryukova K., Catalano C. (2016). Imaging follow-up after liver transplantation. Br. J. Radiol..

[6] Pellerito J.S., Polak J.F. (2019). Introduction to Vascular Ultrasonography E-Book.

[7] García-Criado Á., Gilabert R., Berzigotti A., Brú C. (2009). Doppler Ultrasound Findings in the Hepatic Artery Shortly After Liver Transplantation. Am. J. Roentgenol..

[8] Kimura Y., Tapia Sosa R., Soto-Trujillo D., Kimura Sandoval Y., Casian C. (2020). Liver Transplant Complications Radiologist Can’t Miss. Cureus.

[9] Singh A.K., Nachiappan A.C., Verma H.A., Uppot R.N., Blake M.A., Saini S., Boland G.W. (2010). Postoperative Imaging in Liver Transplantation: What Radiologists Should Know. RadioGraphics.

[10] Caiado A.H.M., Blasbalg R., Marcelino A.S.Z., da Cunha Pinho M., Chammas M.C., da Costa Leite C., Cerri G.G., de Oliveira A.C., Bacchella T., Machado M.C.C. (2007). Complications of Liver Transplantation: Multimodality Imaging Approach. RadioGraphics.

[11] Lisotti A., Fusaroli P., Caletti G. (2015). Role of endoscopy in the conservative management of biliary complications after deceased donor liver transplantation. World J. Hepatol..

[12] Law W., Swensson J., Mayhew M., Zaydfudim V., Khot R. (2025). Imaging and management of complications post biliary-enteric anastomosis. Abdom. Radiol..

[13] Lim C.J., Hong K., Lee J.M., Han E., Hong S., Choi Y., Yi N.-J., Lee K.-W., Suh K.-S. (2021). Clinical usefulness of T1-weighted MR cholangiography with Gd-EOB-DTPA for the evaluation of biliary complication after liver transplantation. Ann. Hepato-Biliary-Pancreat. Surg..

[14] Bofill A., Cárdenas A. (2024). A practical approach to the endoscopic management of biliary strictures after liver transplantation. Ann. Hepatol..

[15] Magro B., Tacelli M., Mazzola A., Conti F., Celsa C. (2021). Biliary complications after liver transplantation: Current perspectives and future strategies. Hepatobiliary Surg. Nutr..

[16] Boraschi P., Donati F., Gigoni R., Volpi A., Salemi S., Filipponi F., Falaschi F. (2010). MR cholangiography in orthotopic liver transplantation: Sensitivity and specificity in detecting biliary complications. Clin. Transplant..

[17] Boraschi P., Donati F., Pacciardi F., Ghinolfi D., Falaschi F. (2018). Biliary complications after liver transplantation: Assessment with MR cholangiopancreatography and MR imaging at 3T device. Eur. J. Radiol..

[18] Wang C.K., Cheng Y.F., Chen C.L., Ou H.Y. (2020). Imaging Diagnosis of Biliary Complications of ABO Incompatibility in Living Donor Liver Transplantation. Transplant. Proc..

[19] Thomaides-Brears H., Lepe R., Banerjee R., Duncker C. (2020). Multiparametric MR mapping in clinical decision-making for diffuse liver disease. Abdom. Radiol..

[20] Miller C., Diago Uso T. (2013). The liver transplant operation. Clin. Liver Dis..

[21] Scatton O., Meunier B., Cherqui D., Boillot O., Sauvanet A., Boudjema K., Launois B., Fagniez P.L., Belghiti J., Wolff P. (2001). Randomized Trial of Choledochocholedochostomy with or Without a T Tube in Orthotopic Liver Transplantation. Ann. Surg..

[22] McElroy L.M., Daud A., Davis A.E., Lapin B., Baker T., Abecassis M.M., Levitsky J., Holl J.L., Ladner D.P. (2014). A meta-analysis of complications following deceased donor liver transplant. Am. J. Surg..

[23] Polak W.G., Porte R.J. (2006). the Sequence of Revascularization in Liver Transplantation: It Does Make A Difference. Liver Transplant..

[24] Iorio P., Vanderbecq Q., Mouhadi S., Arrivé L. (2023). Imaging of the biliary tract. Curr. Opin. Gastroenterol..

[25] Li J., Yu Y., Zhu L., Li Y., He Q. (2020). Magnetic Resonance Imaging versus Computed Tomography for Biliary Tract Intraductal Papillary Mucinous Neoplasm (BT-IPMN): A Diagnostic Performance Analysis. Med. Sci. Monit. Int. Med. J. Exp. Clin. Res..

[26] Donato H., França M., Candelária I., Caseiro-Alves F. (2017). Liver MRI: From basic protocol to advanced techniques. Eur. J. Radiol..

[27] Palmucci S., Roccasalva F., Piccoli M., Fuccio Sanzà G., Foti P.V., Ragozzino A., Milone P., Ettorre G.C. (2017). Contrast-Enhanced Magnetic Resonance Cholangiography: Practical Tips and Clinical Indications for Biliary Disease Management. Gastroenterol. Res. Pr..

[28] Dai H., Yan C., Jia X., Xiao Y., Liang X., Yang C., Liu K., Zeng M. (2025). Comparative evaluation of non-contrast MRI versus gadoxetic acid-enhanced abbreviated protocols in detecting colorectal liver metastases. Insights Imaging.

[29] Ronot M., Nahon P., Rimola J. (2023). Screening of liver cancer with abbreviated MRI. Hepatology.

[30] An J.Y., Peña M.A., Cunha G.M., Booker M.T., Taouli B., Yokoo T., Sirlin C.B., Fowler K.J. (2020). Abbreviated MRI for Hepatocellular Carcinoma Screening and Surveillance. Radiographics.

[31] Samuel D., Martin E.D., Berg T., Berenguer M., Burra P., Fondevila C., Heimbach J.K., Pageaux G.-P., Sanchez-Fueyo A., Toso C. (2024). EASL Clinical Practice Guidelines on liver transplantation. J. Hepatol..

[32] European Association for the Study of the Liver (2018). EASL Clinical Practice Guidelines: Management of hepatocellular carcinoma. J. Hepatol..

[33] Katz L.H., Benjaminov O., Belinki A., Geler A., Braun M., Knizhnik M., Aizner S., Shaharabani E., Sulkes J., Shabtai E. (2010). Magnetic resonance cholangiopancreatography for the accurate diagnosis of biliary complications after liver transplantation: Comparison with endoscopic retrograde cholangiography and percutaneous transhepatic cholangiography - long-term follow-up. Clin. Transpl..

[34] Ethiraju V., Arunachalam V.K., Vijayaragavan P., Poyyamoli S., Kumar R., Rajasekaran S., Mahadevan G.S., Cannane S., Arunachalam P., Ramasamy R. (2023). Liver Transplant Complications—A Pictorial Review. Indographics.

[35] Carbone M., Della Penna A., Mazzarelli C., De Martin E., Villard C., Bergquist A., Line P.D., Neuberger J.M., Al-Shakhshir S., Trivedi P.J. (2023). Liver Transplantation for Primary Sclerosing Cholangitis (PSC) with or Without Inflammatory Bowel Disease (IBD)—A European Society of Organ Transplantation (ESOT) Consensus Statement. Transpl. Int..

[36] Allard R., Smith C., Zhong J., Sheridan M., Guthrie A., Albazaz R. (2020). Imaging post liver transplantation part II: Biliary complications. Clin. Radiol..

[37] Esser H., de Jong I.E.M., Roos F.M., Bogensperger C., Brunner S.M., Cardini B., Dutkowski P., Eker H., Ferreira-Gonzalez S., Forbes S.J. (2025). Consensus classification of biliary complications after liver transplantation: Guidelines from the BileducTx meeting. BJS.

[38] Hassouneh R., Beran A., Rosenheck M., Sosio J., Olchawa N., Kubal C., Ghabril M., Gromski M.A. (2024). Risk factors for biliary strictures and leaks after living-donor liver transplantation: A systematic review and meta-analysis. J. Gastrointest. Surg..

[39] Liu B., Yang J., Wu Y., Chen X., Wu X. (2024). Application of dynamic enhanced scanning with GD-EOB-DTPA MRI based on deep learning algorithm for lesion diagnosis in liver cancer patients. Front. Oncol..

[40] Cai L., Yeh B.M., Westphalen A.C., Roberts J., Wang Z.J. (2017). 3D T2-weighted and Gd-EOB-DTPA-enhanced 3D T1-weighted MR cholangiography for evaluation of biliary anatomy in living liver donors. Abdom. Radiol..

[41] Frydrychowicz A., Lubner M.G., Brown J.J., Merkle E.M., Nagle S.K., Rofsky N.M., Reeder S.B. (2012). Hepatobiliary MR Imaging with Gadolinium Based Contrast Agents. J. Magn. Reason. Imaging.

[42] Argirò R., Sensi B., Siragusa L., Bellini L., Conte L.E., Riccetti C., Del Vecchio Blanco G., Troncone E., Floris R., Salavracos M. (2023). Liver-Specific Contrast-Enhanced Magnetic Resonance Cholangio-Pancreatography (Ce-MRCP) in Non-Invasive Diagnosis of Iatrogenic Biliary Leakage. Diagnostics.

[43] Marth A.A., Auer T.A., Walter-Rittel T.C., Nevermann N., Krenzien F., Schmelzle M., Müller T., Kolck J., Wieners G., Geisel D. (2023). Gd-EOB-DTPA-MRCP to localize bile leakage after liver trauma and surgery: Impact on treatment and outcome. Eur. Radiol..

[44] Bozer A. (2024). Hepatobiliary-Specific MRI Contrast Agent Detection of Subvesical Duct (Luschka’s) Injury Post-Laparoscopic Cholecystectomy: A Case Report. J. Belg. Soc. Radiol..

[45] Kim Y.K., Kim C.S., Lee J.M., Ko S.W., Chung G.H., Lee S.O., Han Y.M., Lee S.Y. (2006). Value of Adding T1-Weighted Image to MR Cholangiopancreatography for Detecting Intrahepatic Biliary Stones. Am. J. Roentgenol..

[46] Romagnuolo J., Bardou M., Rahme E., Joseph L., Reinhold C., Barkun A.N. (2003). Magnetic resonance cholangiopancreatography: A meta-analysis of test performance in suspected biliary disease. Ann. Intern. Med..

[47] Familiari L.T. (2004). MRCP Vs ERCP: A Comparative Study in Diagnosis of Common Bile Duct Stones. Gastrointest. Endosc..

[48] Kumar A., Mohanty N.R., Mohanty M., Dash S. (2023). Comparison of MRCP and ERCP in the evaluation of common bile duct and pancreatic duct pathologies. Front. Med Technol..

